# Chronic Mental Health Sequelae of Climate Change Extremes: A Case Study of the Deadliest Californian Wildfire

**DOI:** 10.3390/ijerph18041487

**Published:** 2021-02-04

**Authors:** Sarita Silveira, Mariah Kornbluh, Mathew C. Withers, Gillian Grennan, Veerabhadran Ramanathan, Jyoti Mishra

**Affiliations:** 1 Department of Psychiatry, University of California, San Diego, CA 92037, USA; sarita.silveira@social.mpg.de (S.S.); ggrennan@ucsd.edu (G.G.); 2 Neural Engineering and Translation Labs, University of California, San Diego, CA 92037, USA; 3 Department of Psychology, University of South Carolina, Columbia, SC 29208, USA; mariahk@mailbox.sc.edu; 4 Department of Psychology, California State University, Chico, CA 95929, USA; mwithers@csuchico.edu; 5 Scripps Institution of Oceanography, University of California, San Diego, CA 92037, USA; vramanathan@ucsd.edu

**Keywords:** climate change, environmental disaster, wildfire, mental health, childhood trauma, resilience, mindfulness

## Abstract

**Introduction.** Weather-related disasters, such as wildfires exacerbated by a rise in global temperatures, need to be better studied in terms of their mental health impacts. This study focuses on the mental health sequelae of the deadliest wildfire in California to date, the Camp Fire of 2018. **Methods.** We investigated a sample of 725 California residents with different degrees of disaster exposure and measured mental health using clinically validated scales for post-traumatic stress disorder (PTSD), major depressive disorder (MDD) and generalized anxiety disorder (GAD). Data were collected at a chronic time-point, six months post-wildfire. We used multiple regression analyses to predict the mental health outcomes based on self-reported fire exposure. Additionally, we included vulnerability and resilience factors in hierarchical regression analyses. **Results.** Our primary finding is that direct exposure to large scale fires significantly increased the risk for mental health disorders, particularly for PTSD and depression. Additionally, the inclusion of vulnerability and resilience factors in the hierarchical regression analyses led to the significantly improved prediction of all mental health outcomes. Childhood trauma and sleep disturbances exacerbated mental health symptoms. Notably, self-reported resilience had a positive effect on mental health, and mindfulness was associated with significantly lower depression and anxiety symptoms. **Conclusion.** Overall, our study demonstrated that climate-related extreme events, such as wildfires, can have severe mental illness sequelae. Moreover, we found that pre-existing stressful life events, resilient personality traits and lifestyle factors can play an important role in the prevalence of psychopathology after such disasters. Unchecked climate change projected for the latter half of this century may severely impact the mental wellbeing of the global population, and we must find ways to foster individual resiliency.

## 1. Introduction

Natural disasters, such as floods, cyclones, droughts and fires have become more intense during the last four decades when the planet has warmed by more than 0.5 °C [1]. With continued emissions, these disasters are predicted to increase as a consequence of climate change [1,2]. The annual western forest-fire area in the US increased by ~1000% from 1984 to 2017 [3]. Since the 1970s to 2018, particularly California has witnessed an eight-fold increase in the areal extent of fires [4,5]. During the same period, California (CA) has warmed by 1.4 °C (about 50% greater than global warming estimates) largely due to anthropogenic sources [1].

Recent studies [5,6] have provided compelling evidence that climate warming has had a definitive role in CA fire extremes. These studies show that year-round warming has increased the aridity of the region due to the exponential increase in evaporation with temperature. The response of the fire extent to aridity is exponential, i.e., warming-driven fuel drying is increasingly impactful. Essentially, climate change is a force-multiplier, accelerating the pre-existing propensity of fires in CA. Between 2017 and 2018 alone, there were approximately 16,000 wildfires reported in this state, with 2 million acres of land burned, resulting in USD 13.7 billion in costs of damages, and ~250 reported injuries, 25% of which resulted in death [7,8].

Weather extremes driven by climate change, such as the California wildfires, are associated with huge costs to human health [9,10,11]. It is projected that the US population exposed to such climate extremes will nearly double by 2050, affecting nearly 25% of all humans in the US each year [12].

Prior studies have stressed the mental health consequences of environmental disasters [10,13,14,15,16]. Yet, we need to better study the mental health impacts in the context of weather disasters exacerbated by climate change. Such disasters could expose specific vulnerabilities and mental health outcomes that may be unique to climate change-driven events and not to routine natural disasters. The main distinction between naturally occurring weather disasters from those amplified by unchecked anthropogenic climate change is that weather disasters forced by climate change will progressively get worse [12].

The two most common adverse mental health outcomes of natural disasters are depression and post-traumatic stress disorder (PTSD) [14]. While research provides evidence for a cumulative impact of exposure to disasters [17,18], most studies report data from the aftermath of a single disaster, and most of those again are classified as natural disasters, unlike recent climate change-driven extreme environmental events. Prior studies of wildfires provide evidence for mental health sequelae, particularly for PTSD symptoms in California residents in the immediate aftermath of the 1991 firestorm [19]. Symptoms were shown to substantially decrease over time [20]. PTSD symptoms were also found in firefighters one month after the occurrence of wildfires in Greece [21]. Yet, to the best of our knowledge, no research to date has addressed distal mental health outcomes, i.e., PTSD, depression and anxiety symptoms in civilians differentially exposed to recent climate change exacerbated wildfires.

A causal pathways framework suggests direct effects of climate change on mental health through exposure to traumatic stressors, as well as indirect effects mediated by impacted physical health, physical environment or community wellbeing [22,23]. Prior research suggests that an adverse impact of environmental disasters is dependent on the degree of disaster exposure. In those studies, degree of exposure was defined objectively (e.g., as geographical distance of residence from the epicenter of the disaster) [24,25] or subjectively (e.g., as degree of household damage) [24]. It has also been shown that PTSD symptoms occur in relation to fire exposure particularly in highly exposed individuals who have experienced loss of residency or injuries [19]. However, it remains an open question whether different degrees of physical and mental exposure differentially impact PTSD, depression and anxiety.

Notably, it has also been argued that the impact of fire exposure on mental health is highly moderated by pre-existing vulnerabilities [26]. Previous research has shown that past-year stressful life events are associated with an increase in current year risk of mental disorders, particularly in adults with adverse childhood experiences [27]. Adversity in the form of child abuse and/or neglect is a transdiagnostic factor that increases risk for several mental disorders [28]. A potential mechanism is stress sensitization, i.e., lower tolerance to stress due to adversities experienced early in life. Yet, prior research in the context of environmental disasters, now transforming into climate change-driven extreme events, has not taken these pre-existing vulnerabilities into account as we do here.

Finally, it is important to identify survivors of serious environmental disasters who have the capacity to mitigate adverse outcomes. Particularly relevant in this context is the notion of resilience, i.e., the ability to recover quickly and adapt well in the face of adversities. Yet, only a few studies to date have investigated dimensions of personal resilience in climate adaptation [20,29]. One personal resiliency characteristic that has been suggested as a pathway towards achieving sustainable climate adaptation is mindfulness, i.e., a non-judgmental attentiveness to the present moment [30]. Additionally, psychological resilience can be conceptualized from a social–ecological angle. Particularly, social support has consistently been highlighted as an indicator of resilience [29]. Specific lifestyle factors may also be protective in the development of psychopathology. While sleep is known to play a crucial role in various mental disorders, recent research increasingly suggests that the relationship between sleep disturbances and symptom severity is bi-directional [31,32]. Altered sleep not only temporally precedes the onset of psychopathology but may also serve as a risk or resilience factor [33,34]. Similarly, the role of physical exercise in preventing stress-related psychopathology is supported by its effects on several neurobiological factors linked to individual resilience, including attenuation of stress responses and increased release of endorphins [35].

In the current study, we aim to understand the mental health sequelae of a serious climate change exacerbated event, specifically, California’s deadliest wildfire to date, the 2018 Camp Fire. Uniquely, we assess mental health in the context of fire exposure, as well as several factors that may serve to impart vulnerability or resilience. Ultimately, we aim to gain some insight towards identifying and supporting individuals who must rebuild their displaced, post-disaster lives.

## 2. Materials and Methods

### 2.1. Sample

**Figure 1:**
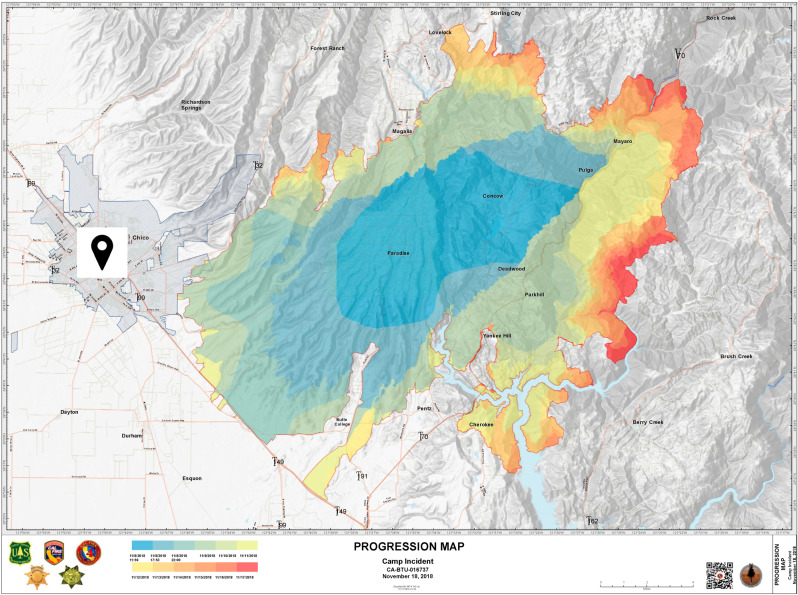
Progression map of the 2018 Camp Fire and location of the study site Chico. Source: Wildfire Today; posted on 18 November 2018.

The study included 725 (mean age: 27.01 ± 13.59; age range: 18–84 years) California residents sampled in spring 2019, six months after the 2018 Camp Fire. Participants were recruited at three sites in California, one in San Diego and two in Chico. While San Diego is at a distance of approximately 600 miles from the center of the Camp Fire and unaffected by the wildfire, Chico is among the cities whose residents were most affected, within 10–15 miles of the center of the Camp Fire (Figure 1). The samples recruited from Chico were either students in the department of psychology at the California State University (CSU) (sample n = 361) or individuals enrolled in the CSU Basic Needs program that provided disaster relief and community-based support directly to Camp Fire victims (sample n = 111) [36]. The sample from San Diego was recruited at the University of California, San Diego (UCSD, sample n = 253). The response rate of invited study participants from the CSU Basic Needs program was 37%; response rates could not be determined for CSU Psychology and UCSD samples as there was no determinable upper limit to the number of individuals who may have seen the recruitment advertisement. Based on these recruitment sites in relation to the Camp Fire, we refer to these groups as “primary proximity and help-seeking” (those in the Basic Needs program), “primary proximity” (CSU students not in the Basic Needs program) and “secondary proximity” (those from San Diego).

The study was approved by the institutional review boards of the University of California, San Diego (IRB#180140) and California State University at Chico (IRB#22838). All study participants provided written informed consent. A sample comparison regarding demographic variables from the three recruitment sites is shown in Table 1.

### 2.2. Measures

Six months after the 2018 Camp Fire, all study participants reported on the following measures. This time of assessment is considered suitable in signifying chronic mental health outcomes [38]. The Research Electronic Data Capture (REDCap; [39]) tool was used for survey administration.

#### 2.2.1. Demographics

Assessed demographic variables included age, sex, race (Caucasian, African American, Pacific Islander, Asian, American Indian or mixed) and socio-economic status (SES). Due to low prevalence of individuals with American Indian or Pacific Islander origin (<1%), we extended the racial category “mixed” to “mixed/other”. SES composite scores were assessed using an adapted version of the family affluence scale [37]. This scale measures family wealth based on family ownership of objects of value (e.g., car/computer) and produces a composite score ranging from 0 (low affluence) to 3 (high affluence). Demographic predictors that have previously been associated with effects of environmental disasters on mental health include sex and SES [14].

#### 2.2.2. Life Events

We evaluated life events related to fire and to childhood adversity. For the former, we used the Life Events Checklist (LEC-5; [40]) from the Diagnostic and Statistical Manual of Mental Disorders (DSM-5), which assesses potentially traumatic events that might have happened at any time in life. It inquires the exposure to events on a 5-point nominal scale (happened to me = 1, witnessed it = 2, learned about it = 3, part of my job = 4, not sure = 5 and does not apply = 0). The scale allows for multiple responses. A vast variety of events are covered, yet we focused on an item assessing fire exposure. Positive responses can be given to multiple levels of exposure, if applicable. Additionally, LEC-5 “witnessed it” responses on fire exposure pertain to real life events and do not include exposure to pictures or videos of the fire in the media. Here, we did not explicitly inquire about exposure to the 2018 Camp Fire as the CSU IRB objected to inclusion of such a direct question given its potential to lead to further traumatic stress.

Additionally, experiences of child maltreatment during the first 18 years of life were assessed using the 28-item brief screening version of the Childhood Trauma Questionnaire (CTQ; [41]). The CTQ is a self-administered retrospective inventory consisting of five categories of child neglect and abuse. Each category entails five items that are rated on a 5-point Likert scale ranging from “never true” (= 1) to “very often true” (= 5).

#### 2.2.3. Mental Health Outcomes

All mental health scales for PTSD, depression and anxiety were assessed by self-report. PTSD symptom severity was measured using the PTSD-Checklist (PCL-5 [42]). The PCL-5 consists of 20 questions that cover experience of PTSD symptoms, such as memories, dreams, avoidance of certain external or internal stimuli. Responses are given on a 5-point Likert scale asking how much one was bothered by these experiences from “not at all (=0)” to “extremely (=4)”. To assess major depressive disorder (MDD), we used the Patient Health Questionnaire (PHQ-9; [43]), a diagnostic instrument that scores each of the 9 DSM-5 criteria for MDD on a 4-point Likert scale assessing frequency of symptoms from “not at all” (=0) to “nearly every day” (=3). To assess Generalized Anxiety Disorder (GAD), we used the 7-item brief scale GAD-7 [44]. Frequency of anxiety symptoms is scored on a 4-point Likert scale from “not at all” (=0) to “nearly every day” (=3).

#### 2.2.4. Resilience Factors

We assessed subjective resilience and lifestyle factors that may impart resiliency, specifically, sleep quality, exercise, mindfulness and emotional support.

The Brief Resilience Scale (BRS; [45]) measures resilience as the capacity to bounce back after tough times, with 6 items on a 5-point Likert scale, half of them inverted from “strongly disagree” (=1) to “strongly agree” (=5).

Sleep quality was assessed with regard to sleep disturbances, using the short form of the Patient-Reported Outcomes Measurement Information System (PROMIS)—Sleep Disturbance scale [46]. It consists of 8 items that assess sleep disturbances (e.g., “my sleep was restless”) in the past week on a 5-point Likert scale ranging from “not at all” (=1) to “very much” (=5).

To investigate physical exercise, we use three questions of the Godin Leisure-Time Exercise Questionnaire [47]. Participants were asked how many times on average during a typical 7-day period they do strenuous, moderate or mild exercise for more than 15 min.

The disposition of being mindful, which can be conceptualized as open and receptive awareness, was measured with the Mindful Attention Awareness Scale (MAAS; original scale by [48], implemented in the version described by [49]). The MAAS entails 14 items, such as “I could be experiencing some emotion and not be conscious of it until sometime later”, that are inversely scored on a 6-point Likert scale from “almost always” (=1) to “almost never” (=6).

Due to its well-documented impact on mental and physical health, we additionally measured emotional support from social relationships using the respective subscale from the NIH Toolbox on Social Relationships (SR) [50]. The sum of 8 items on a 5-point Likert scale from “never” (=0) to “always” (=4) represents the presence and frequency of social support in the participants’ lives.

### 2.3. Data Analysis

#### 2.3.1. Exposure to Fires across Participant Subgroups

We compared the participant subgroups recruited from the three sites in their self-reported degree of exposure to the wildfires. For this, we used the “fire or explosion” life event of the LEC-5. The different types of exposure to fire were dummy coded, and chi-square tests for all five qualitatively different forms of exposure were computed between the three participant pools. Due to the fact that the life event scale allows for multiple responses, each dummy coded variable is treated as independent. As the study sample was recruited from different locations in California, this analysis step is included to confirm that study participants in closer proximity to the outbreak of the fires were indeed more directly exposed.

#### 2.3.2. Prediction of Mental Health Outcomes

We computed separate multiple regressions for each mental health outcome of PTSD, depression and anxiety. Group membership (directly exposed, indirectly exposed and not exposed) together with demographic variables, age, sex, ethnicity and SES, were modeled as predictors. No multicollinearity between independent variables was found as indicated by variance inflation factors (VIF < 3). Due to non-parametric score distributions in the mental health outcomes, we performed bootstrapping using 1000 bootstrap samples. We report three statistical measures of model fit. The F-test for overall model significance indicates whether the regression model fits the data, R^2^ indicates the percentage of explained variance of the outcome and the Akaike information criterion (AIC) estimates the relative model fit and serves as an index for model comparison. Using hierarchical multiple regression analyses, demographics factors were included in the first step, and factors of vulnerability and resilience (i.e., childhood trauma, resilience, sleep quality, exercise, mindfulness and emotional support) were included in a second step. This approach allowed us to test whether vulnerability and resilience factors added value to the prediction of mental health outcomes, as indicated by a significant increase in R^2^ by means of model extension.

For the purpose of group comparisons of mental health status in people with different exposure to fire, we further computed non-parametric Mann–Whitney-U tests. We, therefore, created a variable that groups participants in degrees of fire exposure. In case the participant reported multiple types of exposure, group membership was defined by the highest reported level of exposure (“learned about it” < “witnessed it” < “happened to me”).

For exploratory purposes, we additionally included interaction terms between fire exposure and significant vulnerability and resilience factors that could be linked to mental health outcomes to the model. These exploratory analyses are based on the assumption that vulnerability or resilience factors might mediate an association between fire exposure and mental health.

## 3. Results

### 3.1. Exposure to Wildfires across Participant Subgroups

**Figure 2:**
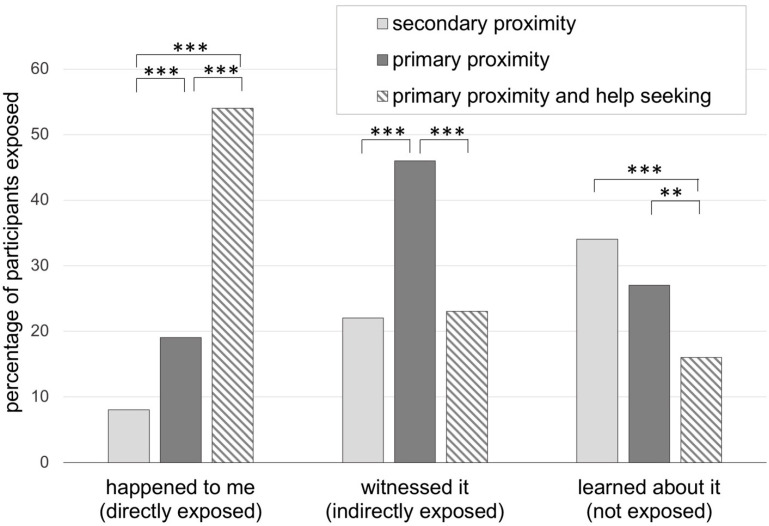
Fire exposure in 725 individuals in primary proximity to the 2018 California Camp Fire, with or without help-seeking behavior, and in secondary proximity to the wildfire. Those responding as fire happened to me, witnessed it or learned about it, respectively, are referred to here as directly exposed, indirectly exposed and not exposed. Chi-square group comparisons, df = 1, ** *p* < 0.01, *** *p* ≤ 0.001.

We confirmed significant differences between participant subgroups in their self-reported exposure to fire, particularly regarding the amount of people that learned about the fires (*χ*^2^ = 11.79, df = 2, n = 725, *p* = 0.003), witnessed the fires (*χ*^2^ = 39.51, df = 2, n = 725, *p* < 0.001) or who reported the fires happening to them directly (*χ*^2^ = 103.92, df = 2, n = 725, *p* < 0.001). While participants with secondary proximity to the Camp Fire mainly reported learning about the fires, participants with primary proximity either witnessed the fires or reported the fire happening to them (i.e., primary proximity and help seeking subgroup) (Figure 2). Additionally, for a very small percentage of participants (1.6% of secondary proximity; 1.7% of primary proximity; 1.8% of primary proximity and help-seeking), exposure to fires was part of their work. Less than 1% of participants in each group were not sure how to classify their fire exposure.

### 3.2. Mental Health Outcomes of Exposure to Wildfires

In the following analyses, we refer to individual positive responses on “LEC-5: happened to me” as “directly exposed”, positive responses on “LEC-5: witnessed it” as “indirectly exposed” and positive responses on “LEC-5: learned about it” as “not exposed”. Results of the bootstrapped multiple regression for mental health outcomes (i.e., PTSD/depression/anxiety) as predicted by sample demographics and fire exposure (directly/indirectly/not exposed) are presented in Table 2. Results of the Mann–Whitney-U group comparisons are presented in Figure 2.

#### 3.2.1. Post-Traumatic Stress Disorder

**Figure 3:**
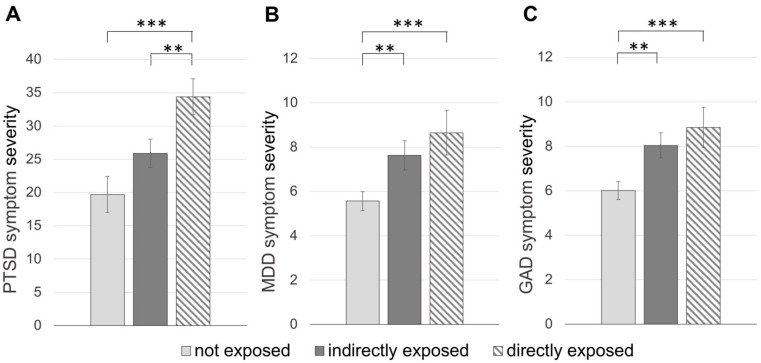
Mental health outcomes in individuals with different degrees of fire exposure (**A**) PTSD: post-traumatic stress disorder, (**B**) MDD: major depressive disorder, (**C**) GAD: generalized anxiety disorder. Means and standard errors are plotted. Group membership was determined by highest level of exposure. Sample size of groups were near equivalent; “not exposed” n = 124, “indirectly exposed” n = 201 and “directly exposed” n = 147. ** *p* < 0.01, *** *p* ≤ 0.001.

The multiple regression model to explain variance in PTSD PCL-5 scores (mean: 26.68 ± 19.50, score range: 0–78) by demographic variables showed overall significance: *F* = 2.52, df_1_ = 10, df_2_ = 153, *p* = 0.007, *R*^2^ = 0.12, AIC = 1596.15. Scores on the PCL-5 were significantly higher in directly exposed individuals (Figure 3A). Lower SES and mixed/other ethnic background were associated with higher PCL-5 scores.

#### 3.2.2. Major Depressive Disorder

The multiple regression model to explain PHQ-9 scores (mean: 6.74 ± 5.94; score range: 0–27) was significant: *F* = 8.53, df_1_ = 10, df_2_ = 564, *p* < 0.001, *R*^2^ = 0.12, AIC = 3908.02. In this model, individuals that were directly and indirectly exposed to fires showed significantly higher scores on the PHQ-9 (Figure 3B). Younger age, lower SES, Caucasian and mixed/other ethnic background were associated with higher MDD symptom severity.

#### 3.2.3. General Anxiety Disorder

The regression model with GAD-7 scores as mental health outcome (mean: 7.09 ± 5.65; score range: 0–21) was significant: *F* = 11.52, df_1_ = 10, df_2_ = 565 *p* < 0.001, *R*^2^ = 0.16, AIC = 3821.05. Both directly and indirectly fire-exposed individuals reported higher symptom severity on the GAD-7 (Figure 3C). Again, younger age, lower SES, Caucasian and mixed/other ethnic background, and additionally male gender, were related to higher scores on this measure.

### 3.3. Influence of Vulnerability and Resilience Factors

#### 3.3.1. Post-Traumatic Stress Disorder

**Figure 4:**
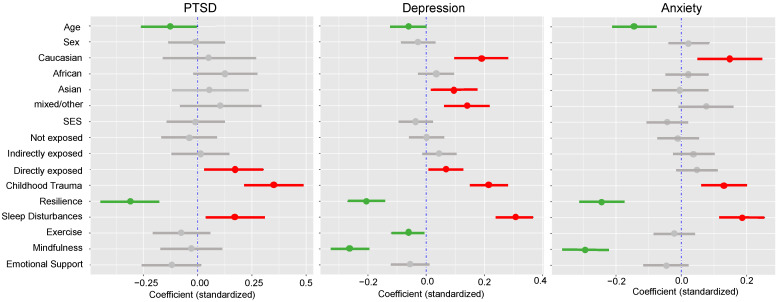
Predictors of mental health for PTSD, depression (MDD) and anxiety (GAD). Standardized model coefficients are plotted with 95% confidence intervals. Significant positive predictors are shown in red, negative predictors in green and non-significant predictors in grey.

The hierarchical regression analyses to predict PTSD PCL-5 scores showed significantly improved model fit when including vulnerability and resilience factors: *R*^2^ change = 0.35, df_1_ = 6, df_2_ = 147, *p* < 0.001, AIC = 1311.02. PCL-5 scores were positively associated with childhood trauma (CTQ) scores and negatively associated with resilience (BRS) scores. Out of the lifestyle factors, only sleep quality was found to predict PCL-5 scores, with higher sleep disturbance (PROMIS) scores related to higher symptom severity. The inclusion of vulnerability and resilience factors in the model changed associations between PCL-5 scores and demographic variables. Younger age was significantly associated with higher scores; however, no associations could be found anymore for SES or ethnic descent (Table 3, Figure 4).

#### 3.3.2. Major Depressive Disorder

Similarly, for MDD symptoms measured on the PHQ-9, the regression model including vulnerability and resilience factors in addition to demographics and fire exposure was significantly improved: *R*^2^ change = 0.43, df_1_ = 6, df_2_ = 558, *p* < 0.001, AIC = 3232.16. Higher childhood trauma scores and higher sleep disturbance scores, as well as lower resilience scores, lower exercise scores and lower mindfulness scores were related to higher PHQ-9 scores. Again, the inclusion of vulnerability and resilience factors changed associations between demographic variables and PHQ-9 scores. An association with SES was not significant; however, higher scores were found in people with Asian descent. Additionally, no significant link could be found between indirect fire exposure and higher scores on the PHQ-9 (Table 3, Figure 4).

#### 3.3.3. Generalized Anxiety Disorder

Adding vulnerability and resilience factors as predictors of anxiety symptoms on the GAD-7 led to an increased model fit: *R*^2^ change = 0.32, df_1_ = 6, df_2_ = 559, *p* < 0.001, AIC = 3277.73. There were significant positive associations with childhood trauma and sleep disturbance scores, and negative associations with resilience and mindfulness scores. Similar to the models on PTSD and depression, the inclusion of vulnerability and resilience factors led to non-significant associations between GAD-7 scores and SES. Additionally, no significant association was found between anxiety scores and mixed or other ethnic background or participant sex. In this model, neither indirect nor direct exposure to fires was associated with higher GAD-7 scores (Table 3, Figure 4).

#### 3.3.4. Interactions between Fire Exposure and Vulnerability or Resilience Factors

Exploratory inclusion of interaction terms between fire exposure, i.e., both indirect and direct exposure, and vulnerability or resilience factors, did not yield any significant results, *p* > 0.05.

## 4. Discussion

The current study investigates mental health outcomes of a significant environmental disaster, the deadliest California wildfire in history, the Camp Fire of 2018, which has been shown to be driven by climate change-induced temperature extremes [5,6]. Our analyses include three groups of participants that were identified by proximity as well as help-seeking behavior post-fire. Previous research suggests an association between mental health outcomes after disasters and the degree of exposure as measured in distance to the disaster, degree of household damage or physical injury [19,24,25]. In the context of this wildfire, we confirmed whether our cohort was directly exposed (i.e., to whom the fire happened), indirectly exposed (i.e., who witnessed the fire) or not exposed (i.e., who learned about the fire) as per their self-reported fire exposure. With regard to mental health outcomes, significantly higher symptom scores on mental health outcomes were found in directly exposed individuals, particularly for PTSD and depression. These findings align with previous research showing similar short- and long-term impacts of disaster exposure on mental health [51,52].

PTSD, depression and anxiety disorders can all be conceptualized as stress-related disorders for which environmental stressors and individual (biological and psychological) stress responses are central to pathogenesis. In line with this, not only the experience of wildfires but also childhood trauma (i.e., child abuse and neglect) was consistently found to increase risk of all of these symptoms. This may be particularly relevant in the study samples from Chico, where over 70% of the population are reportedly affected by childhood adversities [53]. Our findings support the notion that adverse childhood experiences may serve as a vulnerability factor for mental health sequelae in adulthood [28]. Similarly, lower socio-economic status was also initially identified as a consistent predictor of PTSD, depression and anxiety symptoms, aligned with prior research on climate hazards to human health [10]. However, this association between mental health and socio-economic status was only significant when childhood adversities as well as other cognitive and lifestyle factors were not accounted for.

We further included measures of resilience and resiliency-imparting lifestyle in our study. Several factors, including personal, social, economic, institutional, infrastructure and community resources, may contribute to resilience and adaptation to environmental disaster, and can be used as indicators thereof. Our study focuses on personal resilience as the ability to bounce back after stressful life events, as well as sleep quality, physical exercise, mindfulness and emotional support, which may all contribute to resilience. As hypothesized, we consistently found self-reported resilience to be inversely associated with PTSD, depression and anxiety symptom severity. Further, higher levels of sleep disturbances were related to higher scores on all clinical symptom scales. These results highlight the relevance of sleep in mental health, although our analyses cannot disentangle whether sleep disturbances were symptomatic of the mental disorders or a risk factor to their development [32].

Additionally, mood disorders of depression and anxiety were negatively associated with mindfulness. It has been argued that mindfulness may support a fundamental shift in the way we think about and act on local and global ecological crises [54], and thus plays a role in developing psychological resilience. Recent evidence shows that mindfulness correlates with responses to severe climate events, recovery and proactive climate adaptation [55]. Although trait mindfulness is considered to relate to greater psychological adjustment following exposure to trauma in general, we did not find an effect of mindfulness on PTSD symptoms. Similarly, a study in Tsunami disaster survivors did not find a positive effect of trait mindfulness on PTSD symptoms, indicating that mindfulness may not be a protective factor against post-traumatic stress in trauma-exposed individuals [56]. Much interventional research shows that mindfulness can be enhanced and serve to provide positive mental health benefits, which should be encouraged in these vulnerable communities [57,58,59,60]. Finally, physical exercise was found to only have a positive effect on depression symptom severity. While we found no positive effect of emotional support on any of the mental health outcomes, future work with a focus on different socio-emotional protective factors may further elucidate the role of family and community in coping with mental health impacts.

A limitation of this study is an inhomogeneity of socio-demographic characteristics between study participants that were recruited at different sites, and thus a lack of representativeness of samples. While samples were selected with respect to the degree of proximity to and affliction by the 2018 Camp Fire, differences in socio-demographic characteristics may have introduced biases. Particularly, family affluence as an indicator of SES may be directly associated with the degree of fire exposure (e.g., by a loss of family belongings due to the fire) and may thus have skewed subjects in the primary proximity and help-seeking sample with high prevalence of direct fire exposure to lower SES. However, with variance inflation factors of <3 for all independent variables, multicollinearity between fire exposure and socio-demographic variables, and thereby confounding effects between those variables can be widely ruled out. Additionally, Cohen’s *f*^2^ indicated unique effects, particularly of fire exposure on explained variance in outcome measures.

## 5. Conclusions

Our primary finding is that climate-related extremes, such as fires, are significantly associated with sequelae of severely impacted mental wellbeing. With respect to fire disasters, mental health outcomes are dependent on the degree of fire exposure, as well as pre-existing vulnerabilities, particularly due to childhood adversities, and quality of sleep. Trait resilience is associated with a significant reduction in mental health impacts. Mindfulness can serve as a protective factor for depression and anxiety, while exercise shows a buffering effect in the case of depression. This implies that bolstering skills in resilience can be an effective tool in urgently needed disaster relief efforts and programs [61]. The ways in which we respond to environmental disasters exacerbated by climate change pose a complex global sustainability challenge. Proactive climate adaptation requires a set of resilient personality traits and lifestyle factors that preserve mental health and facilitate a much-required cultural shift towards sustainability.

These findings have important implications for societal adaptation to future climate variability. The planet has already warmed by 1 °C since the early twentieth century. In the absence of urgent mitigation steps to curb carbon emissions, the warming is projected to increase four-fold to 4 °C, exposing almost 80% of the world population to extremes such as heat waves. Our findings call for a new focus on monitoring the mental health effects of climate extremes and for developing scalable and sustainable personal remedial measures to cope with these inevitable extremes.

## Figures and Tables

**Table 1:** Sample demographics across all study sites.

Variable	Category	Primary ProximityHelp-Seeking	Primary Proximity	Secondary Proximity	*χ*^2^/*H*	*df*	n
		M ± SD n (%)	M ± SD n (%)	M ± SD n (%)			
Age		27.41 ± 9.72	21.72 ± 3.53	34.40 ± 19.41	93.79 ***	2	725
Sex	Male	28 (25.2)	72 (19.9)	102 (40.3)	33.19 ***	4	725
	Female	82 (73.9)	288 (79.8)	151 (59.7)			
	n/a	1 (0.9)	1 (0.3)				
Ethnicity	Caucasian	77 (69.4)	201 (55.7)	130 (51.4)	100.50 ***	12	725
	African American	3 (2.7)	13 (3.6)	3 (1.2)			
	Asian	7 (6.3)	18 (5.0)	72 (28.5)			
	mixed/other	13 (11.7)	54 (14.9)	30 (11.9)			
	n/a	11 (9.9)	75 (21.0)	18 (7.1)			
SES		1.79 ± 0.63	2.11 ± 0.73	2.31 ± 0.66	40.63 ***	2	713
	n/a	3 (2.7)	5 (1.4)	4 (1.6)			

Note. n/a = unknown or not reported, SES = socio-economic status as indicated by the family affluence scale [37] (range: 0–3). *χ*^2^ = Chi-Square statistics (for categorical variables)/H = Kruskal–Wallis H statistics (for numeric variables) derived from non-parametric group comparisons. *** *p* < 0.001. Df and sample size n correspond to group comparisons; n of Kruskal–Wallis test for SES excludes subjects with missing data.

**Table 2:** Mental health prediction by demographics and fire exposure.

	PTSD				MDD				GAD			
			*CI*				*CI*				*CI*	
	*β*	*f* ^2^	LL	UL	*β*	*f* ^2^	LL	UL	*β*	*f* ^2^	LL	UL
Age	0.03	0.00	−0.16	0.29	−0.07 *	0.03	−0.10	−0.04	−0.10 *	0.07	−0.12	−0.08
Sex (male)	2.96	0.00	−3.09	8.09	0.32	0.00	−0.72	1.30	0.99 *	0.01	0.12	1.92
Caucasian	3.76	0.01	−3.73	11.21	2.10 *	0.01	0.76	3.33	1.54 *	0.00	0.30	2.77
African	13.56	0.02	−5.01	36.02	1.84	0.00	−1.50	5.57	1.01	0.00	−3.04	5.67
Asian	7.99	0.02	−2.06	17.86	0.75	0.00	−0.76	2.31	−0.59	0.00	−2.11	0.89
mixed/other	10.34 *	0.03	0.86	20.19	3.44 *	0.02	1.74	5.12	2.44 *	0.01	0.85	4.17
SES	−5.03 *	0.04	−8.72	−1.31	−1.20 *	0.01	−1.81	−0.60	−1.04 *	0.01	−1.62	−0.42
Not exposed	−3.88	0.01	−9.60	2.06	−0.26	0.00	−1.19	0.63	−0.40	0.00	−1.24	0.44
Indirectly exposed	1.95	0.01	−4.02	8.13	1.49 *	0.01	0.48	2.44	1.24 *	0.01	0.26	2.08
Directly exposed	9.54*	0.06	3.91	15.53	1.99 *	0.02	0.76	3.06	1.62 *	0.01	0.53	2.65

Note. PTSD = post-traumatic stress disorder, MDD = major depressive disorder, GAD = general anxiety disorder, *β* = regression weight, *f*^2^ = effect size (0.02 small, 0.15 medium, 0.35 large), CI = 95% confidence interval, LL = lower limit, UL = upper limit. * statistical significance.

**Table 3:** Mental health prediction by inclusion of vulnerability and resilience factors.

	PTSD				MDD				GAD			
			*CI*				*CI*				*CI*	
	*β*	*f* ^2^	LL	UL	*β*	*f* ^2^	LL	UL	*β*	*f* ^2^	LL	UL
Age	−0.19*	0.02	−0.35	−0.03	−0.03 *	0.01	−0.04	−0.01	−0.06 *	0.03	−0.08	−0.04
Sex (male)	−0.31	0.00	−5.38	5.15	−0.37	0.00	−1.07	0.36	0.29	0.00	−0.40	1.00
Caucasian	2.12	0.00	−4.53	8.88	2.25 *	0.03	1.27	3.18	1.68 *	0.02	0.74	2.71
African	12.26	0.02	−1.55	27.65	1.36	0.00	−3.02	5.11	0.77	0.00	−4.16	5.12
Asian	3.58	0.00	−5.77	13.57	1.56 *	0.01	0.48	2.57	−0.07	0.00	−1.16	1.08
mixed/other	5.85	0.01	−4.05	16.13	2.38 *	0.02	1.04	3.73	1.25	0.01	−0.23	2.69
SES	−0.26	0.01	−4.03	3.33	−0.30	0.00	−0.74	0.20	−0.34	0.00	−0.84	0.17
Not exposed	−1.66	0.00	−6.66	3.32	0.00	0.00	−0.72	0.67	−0.14	0.00	−0.83	0.58
Indirectly exposed	0.48	0.00	−4.20	5.40	0.54	0.00	−0.18	1.28	0.44	0.00	−0.30	1.18
Directly exposed	6.97 *	0.04	1.53	12.50	0.94 *	0.01	0.11	1.81	0.64	0.00	−0.31	1.56
Childhood Trauma	9.04 *	0.22	5.38	12.64	2.00 *	0.13	1.28	2.76	1.17 *	0.03	0.48	1.85
Resilience	−1.22 *	0.16	−1.73	−0.69	−0.25 *	0.14	−0.32	−0.17	−0.28 *	0.12	−0.35	−0.20
Sleep Disturbances	0.54 *	0.05	0.07	1.01	0.27 *	0.23	0.21	0.33	0.16 *	0.05	0.10	0.22
Exercise	−0.03	0.01	−0.07	0.03	−0.01 *	0.02	−0.01	−0.00	−0.00	0.01	−0.01	0.00
Mindfulness	−0.59	0.01	−3.65	2.16	−1.57 *	0.16	−1.96	−1.21	−1.67 *	0.12	−2.08	−1.27
Emotional Support	−0.29	0.00	−0.64	0.06	−0.05	0.04	−0.09	−.00	−0.04	0.01	−0.09	0.03

Note. PTSD = post-traumatic stress disorder, MDD = major depressive disorder, GAD = general anxiety disorder, *β* = regression weight, *f*^2^ = effect size (0.02 small, 0.15 medium, 0.35 large), CI = 95% confidence interval, LL = lower limit, UL = upper limit. * statistical significance.

## Data Availability

The datasets used and/or analyzed during the current study are available from the corresponding author on reasonable request.
